# Thermal Analysis of Crystallization and Phase Transition in Novel Polyethylene Glycol Grafted Butene-1 Copolymers

**DOI:** 10.3390/polym11050837

**Published:** 2019-05-08

**Authors:** Chuanbin An, Yulian Li, Yahui Lou, Dongpo Song, Bin Wang, Li Pan, Zhe Ma, Yuesheng Li

**Affiliations:** 1 Tianjin Key Laboratory of Composite and Functional Materials, and School of Materials Science and Engineering, Tianjin University, Tianjin 300072, China; ancb0306@tju.edu.cn (C.A.); liyulian@tju.edu.cn (Y.L.); yhlou@tju.edu.cn (Y.L.); dongpo.song@tju.edu.cn (D.S.); binwang@tju.edu.cn (B.W.); lilypan@tju.edu.cn (L.P.); ysli@tju.edu.cn (Y.L.); 2 Collaborative Innovation Center of Chemical Science and Engineering (Tianjin), Tianjin 300072, China

**Keywords:** butene-1 copolymer, long-chain graft, crystallization kinetics, melting, phase transition

## Abstract

Copolymerization is an effective strategy to regulate the molecular structure and tune crystalline structures. In this work, novel butene-1 copolymers with different polyethylene glycol (PEG) grafts (number-average molecular weight *M*_n_ = 750, 2000, and 4000 g/mol) were synthesized, for the first time introducing long-chain grafts to the polybutene-1 main chain. For these PEG-grafted copolymers, crystallization, melting, and phase transition behaviors were explored using differential scanning calorimetry. With respect to the linear homopolymer, the incorporation of a trimethylsilyl group decreases the cooling crystallization temperature (*T*_c_), whereas the presence of the long PEG grafts unexpectedly elevates *T*_c_. For isothermal crystallization, a critical temperature was found at 70 °C, below which all polyethylene glycol-grafted butene-1 (PB-PEG) copolymers have faster crystallization kinetics than polybutene-1 (PB). The subsequent melting process shows that for the identical crystallization temperature, generated PB-PEG crystallites always have lower melting temperatures than that of PB. Moreover, the II-I phase transition behavior of copolymers is also dependent on the length of PEG grafts. When form II, obtained from isothermal crystallization at 60 °C, was annealed at 25 °C, PB-PEG-750, with the shortest PEG grafts of *M*_n_ = 750 g/mol, could have the faster transition rate than PB. However, PB-PEG-750 exhibits a negative correlation between transition rate and crystallization temperature. Differently, in PB-PEG copolymers with PEG grafts *M*_n_ = 2000 and 4000 g/mol, transition rates rise with elevating crystallization temperature, which is similar with homopolymer PB. Therefore, the grafting of the PEG side chain provides the available method to tune phase transition without sacrificing crystallization capability in butene-1 copolymers.

## 1. Introduction

Polybutene-1 (PB-1) is one class of polymeric material which has outstanding mechanical properties, such as excellent creep resistance, good crack resistance, good heat resistance, and high hardness [1,2,3,4,5,6,7,8]. There are three possible crystal modifications in PB-1, which are the hexagonal form I/I’, tetragonal form II, and orthorhombic form III [9,10,11]. From the thermodynamic point of view, the hexagonal phase is most stable, of which form I’ is obtained directly by melt or solution crystallization, and form I is converted from the metastable tetragonal form II through solid-solid phase transformation [12,13,14]. Although tetragonal form II is metastable, it is the crystal modification that is usually generated from melt crystallization due to the kinetic advantage [15,16]. In this case, a phase transformation spontaneously happens from the initially generated form (form II) into the stable form (form I), even under quiescent conditions at room temperature [2,17,18]. The crystallites affected by such a phase transition are of great importance to product properties. On one hand, after II-I phase transition, the melting temperature is elevated and the mechanical strength of hardness is improved. On the other hand, when transformation is completely suppressed, form II exhibits a very high fracture strain of over 1000% [19]. Therefore, control of crystallite modification is crucial to material performance [2,5,20].

In addition to the physical means to tune the rate of phase transition [1,21,22,23,24,25,26,27], the chemical regulation of macromolecular structure is another efficient method [28,29,30,31,32]. The molecular structure can be designed by varying molecular weight, as well as regularity, or incorporating the extra structural co-units. Recently, Qiao et al. studied the correlation of the II-I phase transition with molecular weights in a polybutene-1 homopolymer [12,33,34]. It was found that high molecular weight facilitates the formation of the intercrystallite links because of the relative large radius of gyration, with respect to the stacking size of crystalline and amorphous domains, which enhances stress transfer into the lamellae. De Rosa et al. studied the effect of regularity on II-I phase transition [35,36]. Their results demonstrated that *rr* triad defects can significantly promote the phase transition rate and even induce the appearance of hexagonal form I’ directly from amorphous melt.

Copolymerization is also widely used to regulate molecular structure due to the diversity of comonomer types. In the 1960s, Jones studied the influence of α-olefins with no more than 18 carbon atoms on the phase transition of copolymers [31]. It was found that the relatively short comonomers, including ethylene, propylene, and pentene, were able to accelerate phase transition, and the relatively long α-olefin, with 6–18 carbon atoms, slows down phase transition. Zheng et al. incorporated the branched 4-methyl-1-pentene (4M1P) co-units into the polybutene-1 main chain and found that 7.8 mol% 4M1P co-units can completely suppress the occurrence of the II-I phase transition for their experimental duration of four months [19]. Moreover, He et al. utilized 1,5-hexadiene as the comonomer and reported that the incorporated ring-like structural units can also accelerate phase transition [37].

However, the presence of extra co-units may reduce crystallization capability due to the disturbed regularity of the main chain. For example, in the above butene-1/4M1P copolymers, the increase of 4M1P co-units from 0.18 to 12.3 mol% significantly decreases the crystallization temperature from 73.4 to 25.5 °C, with cooling at 5 °C/min [19]. For this problem, a long-chain branch (LCB) provides a potential solution [38,39,40,41,42]. Takamura et al. reported that the introduction of a LCB can significantly accelerate crystallization kinetics [38]. Wang et al. recently prepared a series of long-chain branched polylactide (PLA) and found that, with respect to linear PLA, the presence of LCB increases the nucleation density but reduces the linear growth rate [40]. Inspired by these studies, we wonder whether such long branches can be used in butene-1 copolymers to tune phase transition without compromising the crystallization of the polymer. In actuality, Tarallo et al. studied the crystallization of butene-1/hexene copolymers. Their results show that the increase of hexene concentration from 3.3 to 11.2 mol% decreases the crystallization temperature by more than 20 °C [28]. Apparently, this conjecture does not work for the short branches like hexane. For longer grafts, the specific influences on PB-1 crystallization and phase transition remain mysterious.

In this work, we aim to explore the correlations between relatively long grafts with crystallization and phase transition behaviors. With the reaction intermediate method, long polyethylene glycol (PEG) grafts were successfully introduced to a polybutene-1 backbone. Three PEG grafts, with number-average molecular weights of *M*_n_ = 750, 2000, and 4000 g/mol, respectively, were utilized to vary the length grafts, which kept the graft density identical. For these model copolymers, we explored the (non-isothermal and isothermal) crystallization and melting behaviors. Moreover, we also report that variation of the PEG graft length is an effective approach to tune the phase transition from tetragonal form II to hexagonal form I for polybutene-1 copolymers.

## 2. Materials and Methods

### 2.1. Materials

**Scheme 1:**
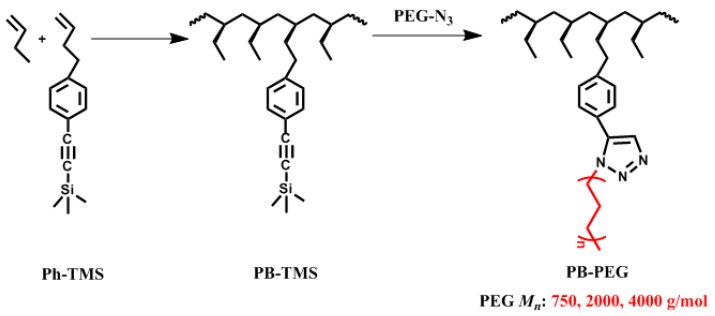
Synthetic route of butene-1/4-[4-(trimethylsilylalkynyl)-phenyl]-1-butylene (Ph-TMS) copolymer and polyethylene glycol (PEG)-grafted butene-1 copolymers.

All synthetic experiments involving air- and/or moisture-sensitive materials were conducted in an MBraun glovebox or by using the standard Schlenk technique under a nitrogen atmosphere. The {Me_2_Si(2,5-Me_2_-3-(2-MePh)cyclopento[2,3-b]thiophen-6-yl)_2_}ZrCl_2_ (rac:meso = 1:1) precatalyst and poly(ethylene glycol) azide at one end (PEG-N_3_) were synthesized according to the literature [43,44,45]. Toluene was used after purification by an Etelux solvent treatment system. The butene-1 gas and ultra-high-purity nitrogen were purchased from the Tianjin Liufang Industrial Gas Distribution Co., Ltd. The triisobutyl aluminum (Al(^i^Bu)_3_, 1.1 mol/L in toluene), trityltetrakis(pentauorophenyl)borate ([Ph_3_C][B(C_6_F_5_)_4_]) cocatalyst, cuprous bromide (CuBr), pentamethyldiethylenetriamine (PMEDTA), and tetrabutylammonium fluoride (TBAF) were obtained from J&K China Chemical Ltd. (Beijing, China) and were used directly without further purification. The synthetic route of the polybutene-1 copolymer and PEG-grafted polybutene-1 is shown in Scheme 1.

### 2.2. Copolymerization of Butene-1 with Ph-TMS

The process of the polymerization reaction is strictly required to be water and air free. The whole reaction system had a capacity of 90 mL. The butene-1 gas (ambient pressure) was fed into the Schlenk flask (150 mL) while the comonomer (0.4 mL) and Al(^i^Bu)_3_ (1.5 mL) were carefully injected into the system. Then, an appropriate amount of toluene was added into the flask to maintain the entire system volume at 70 mL. Next, the solution was mechanical stirred at 400 rpm for 5 min at room temperature to ensure that the butene-1 formed a saturated solution in the presence of toluene. The catalyst (15 µmol) and cocatalyst (30 µmol) were dissolved separately with 20 mL of toluene and rapidly added into the system. Immediately, the rotation speed was increased to 900 rpm and kept constant for 6 min. The pressure of the butene-1 gas throughout the reaction process was maintained using a rubber balloon. Copolymerization was terminated by removing the source of butene-1 gas and injecting 2 mL of MeOH. The reaction solution was poured into an appropriate amount of ethanol to afford the precipitation of the copolymer. This copolymer was re-dissolved in toluene and precipitated with large amounts of ethanol to remove the unreacted monomer and residual catalyst. Finally, the butene-1/Ph-TMS (PB-TMS) copolymer was obtained by drying in a vacuum oven at 40 °C for 24 h to a constant weight (15.4 g). NMR spectra confirmed the successful synthesis of the copolymer (Figure S1a).

### 2.3. Synthesis of Polybutene-1 Grafted with Polyethylene Glycol

The grafted polybutene-1, with different molecular weights of PEG, was prepared via a process which requires strict control of the anhydrous and oxygen-free environment. Grafting experiments of PEG with different molecular weights were carried out in a similar manner. The experiment is described by taking PEG with a molecular weight of 750 g/mol as an example. The copolymer (1.43 g) was dissolved in ultra-dry toluene (70 mL) at 70 °C. A trace amount of TBAF (9 mol% of the total monomer content, 0.02 mL) was placed in the reactor as a catalyst to remove trimethylsilyl groups. After 6 h of reaction, PEG-N_3_ (0.262 g), CuBr (0.0245 mg) and PMEDTA (0.21 mL) were added to the reaction system in sequence. After reacting at 70 °C for 12 h, the polymer was precipitated in cold n-hexane. The obtained polymer was washed with acetone three times. The final PEG-grafted polybutene-1 (1.52 g) was obtained after drying in vacuum at 40 °C for 24 h. NMR results proved that the grafting reaction was complete (Figure S1b).

### 2.4. Methods

#### 2.4.1. Nuclear Magnetic Resonance (^1^H-NMR) and Gel Permeation Chromatography (GPC) Characterization

The ^1^H-NMR characterizations were performed with a Bruker 400 MHz spectrometer. All samples were tested at 120 °C with C_2_D_2_Cl_4_ as a solvent. From the ^1^H-NMR results, the Ph-TMS co-unit incorporations were determined using the formula Ph-TMS mol% = [8*I*_7.39–7.51 ppm_/(2*I*_0.58–1.83 ppm_ − 7*I*_7.39–7.51 ppm_)] × 100%, where *I*_x ppm_ is the peak integral of a proton at a certain (x) ppm. The basic molecular information, including the molecular weights and molecular weight distributions, was obtained at 150 °C using a PL-GPC 220 high-temperature gel permeation chromatography (Figure S2). The measured molecular characteristics of the polybutene-1 homopolymer, butene-1/Ph-TMS copolymer, and PEG-grafted polybutene-1 are summarized in Table 1.

#### 2.4.2. Differential Scanning Calorimetry (DSC)

Differential scanning calorimetry (DSC) measurements were carried out with DSC Q2000 (TA instrument) under a nitrogen atmosphere. Three thermal protocols were employed in this work, in which the sample was first annealed at 180 °C for 10 min to clean the thermal history. Then, the crystallization ability was examined by dynamic cooling experiments at 10 °C /min (Figure S3a). In addition, the isothermal crystallizations were performed at various temperatures to reveal the correlation between crystallization kinetics and molecular structures (Figure S3b).

To explore the II-I phase transition, form II was prepared by isothermal crystallization at different temperatures, which was followed by 50 h annealing at 25 °C (Figure S3c). At last, a heating process was applied at 10 °C/min to examine the generated crystallites.

#### 2.4.3. Fourier Transform Infrared Spectroscopy (FTIR)

A Nicolet 6700 Fourier transform infrared (FTIR) spectrometer with a resolution of 4 cm^−1^ was employed to characterize the modification of the PB-1 crystallites. For the FTIR experiments, the samples were first compressed into thin films of around 200 μm. Then, a Linkam hot stage was used to prepare the crystallites, employing the same thermal protocol as with the DSC measurements.

## 3. Results and Discussion

### 3.1. Non-Isothermal Crystallization

**Figure 1:**
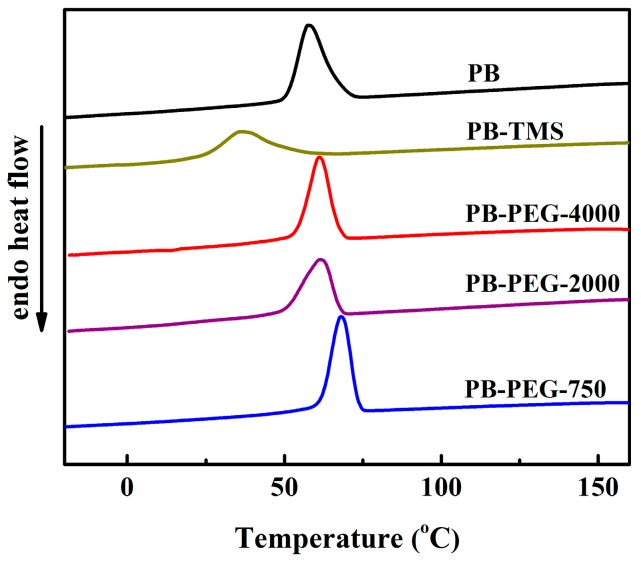
Differential scanning calorimetry (DSC) cooling curves of the homopolymer polybutene-1 (PB), copolymer PB-TMS, and polyethylene glycol (PEG)-grafted butene-1 copolymers.

First of all, the effects of TMS branch and PEG graft on copolymer crystallization were investigated with the DSC dynamic cooling protocol, illustrated by Figure S3a. From the results of Figure 1, it can be seen that due to the disturbed chain regularity, the incorporation of a TMS branch lowers the crystallization temperature (*T*_c_) from 58.3 °C in linear PB to 36.5 °C in the PB-TMS copolymer. However, it is interesting to observe that when the TMS branch was replaced by the long PEG grafts, *T*_c_ was elevated significantly, although the PB-TMS and PB-PEG copolymers have the same branch density. Moreover, the enhancement amplitude of crystallization by PEG is so high that the crystallization temperature of the PB-PEG copolymers becomes higher than that of PB. Table 2 summarizes the values of *T*_c_ as a function of PEG length. Clearly, PB-PEG-750 has the highest crystallization temperature of 68.1 °C, which is around 32 °C higher than PB-TMS. As the length of PEG graft was increased in PB-PEG-2000 and PB-PEG-4000, the crystallization temperatures are slightly decreased to around 63 °C, which is still higher than the copolymer PB-TMS. It can be concluded that long PEG grafts with *M*_n_ between 750 and 4000 g/mol have a distinct influence in elevating the cooling crystallization temperature with respect to PB-TMS.

**Figure 2:**
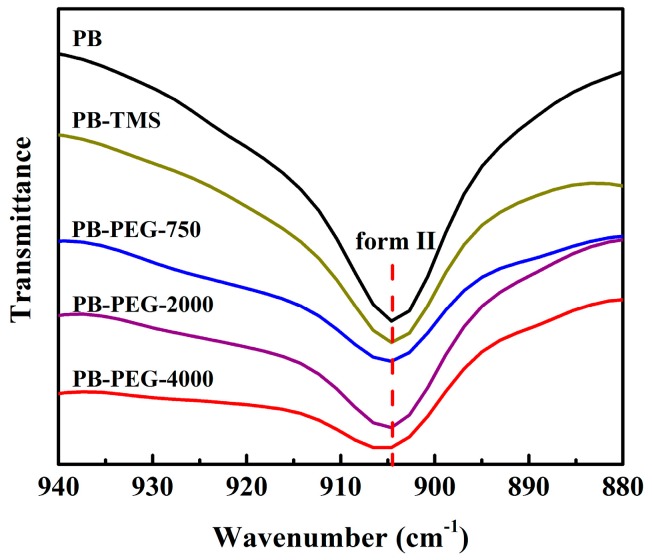
Fourier transform infrared spectroscopy (FTIR) spectrum of the PB, PB-TMS, and PB-PEG copolymers crystallized during cooling at 10 °C/min.

It has been reported that the copolymerization of structural co-units to the polybutene-1 main chain may vary the crystallization polymorphism from the regular form (tetragonal form II) to the thermodynamically stable hexagonal form I’ [37,46]. It is also known that the former has a much faster growth rate than the latter [47]. Then, concerning the aforementioned increased *T*_c_ in PEG-grafted copolymers, the modification of cooling-generated crystallites should be examined to know whether the contribution arises from the variation of crystallization polymorphism. Figure 2 shows the FTIR results for the PB, PB-TMS and PB-PEG copolymers after cooling to room temperature. Clearly, a single absorption band is observed at the wavenumber of 904 cm^−1^, which corresponds to tetragonal form II [48]. There is no signal identified at 925 cm^−1^, which is the characteristic band for polybutene-1 hexagonal modification [48]. Then, it can be concluded that variation of crystallization polymorphism does not contribute to above affected crystallization temperature.

Based on the observation that pure form II was generated, the degree of crystallinity (*X_c_*) is assessed using the following equation:

(1) $$X_{c}=\frac{\Delta H_{m}}{\Delta H_{II}^{o}}\;\times \;100\%$$

where $\Delta H_{m}$ is the melting enthalpy of crystallized form II and $\Delta H_{II}^{o}$ is the melting enthalpy of the pure form II crystal (62 J/g) [49]. The determined values of *X*_c_ are shown in Table 2. Compared with *X*_c_ = 41% in PB, incorporation of the TMS group decreases the crystallinity to 29.3%. Interestingly, in the PB-PEG copolymers, the crystallinity was around 39%, which is very close to linear PB. These results show the featured influence of PEG grafts on crystallization, which increase the cooling crystallization temperature but do not largely reduce the final degree of crystallinity.

### 3.2. Isothermal Crystallization Kinetics

**Figure 3:**
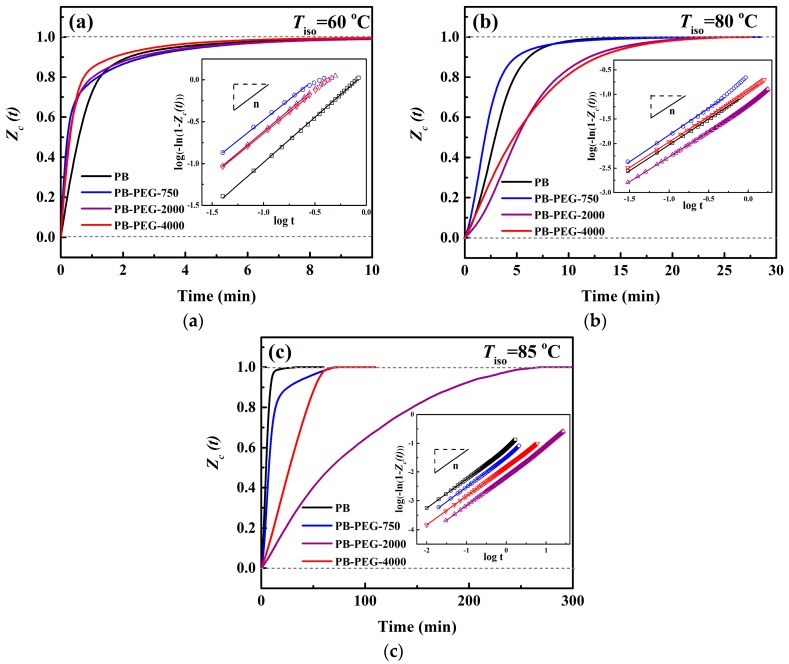
Kinetics of isothermal crystallization at temperatures of (**a**) 60, (**b**) 80, and (**c**) 85 °C.

In reality, crystallization can happen in a broader temperature range than the above cooling crystallization range. To understand the correlation between molecular factors and crystallization kinetics more comprehensively, isothermal crystallization was performed for the PB and PB-PEG copolymers at temperatures ranging from 50 to 85 °C. The crystallization kinetics were quantified by the normalized crystallinity index (*Z_c_*) using the following equation:

(2) $$Z_{c}\left(t\right)=\frac{\Delta H_{m}\left(t\right)}{\Delta H_{\infty }}$$

where $\Delta H_{m}\left(t\right)$ is the integrated enthalpy over the total isothermal period of *t* and $\Delta H_{\infty }$ is the enthalpy of the complete crystallization. Figure 3 displays the crystallization kinetics of the linear PB and grafted PB-PEG copolymers at three representative temperatures of 60, 80, and 85 °C (the results of other temperatures are given in Figure S4). It was unexpected to observe that the PB and PB-PEG copolymers have different temperature dependences of crystallization kinetics. At the relatively low temperature of 60 °C, all PB-PEG copolymers have faster kinetics than PB, which is consistent with the increased *T*_c_ shown in Figure 1. At an elevated temperature of 80 °C, the crystallization of PB becomes faster than that of PB-PEG-2000 and PB-PEG-4000, but still slower than PB-PEG-750. As temperature was further increased to 85 °C, the crystallization kinetics of PB exceed all of the PB-PEG copolymers. The isothermal crystallization kinetics were analyzed using the Avrami model that can be described by the modified expression $\log\left[-\ln\left(1-Z_{c}\left(t\right)\right)\right]=\log k+n\log t$, with the exponent number *n* and rate constant *k*. The insets of Figure 3 show the Avrami plots of crystallization in the homopolymer and PEG-grafted copolymers, in which the slope corresponds to the value of *n*. The quite close values of exponent number *n* suggest that the introduction of long PEG grafts do not vary the crystallization dimensionality.

**Figure 4:**
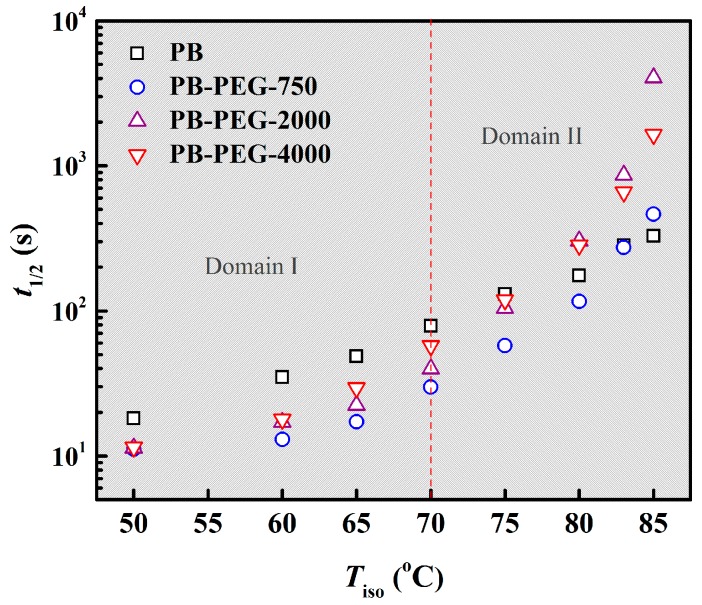
Half-crystallization time (*t*_1/2_) of PB and the PB-PEG copolymers as a function of isothermal crystallization temperatures.

Figure 4 shows the determined half-crystallization time (*t*_1/2_) for the PB and PB-PEG copolymers. Clearly, crystallization is slowed down with increasing temperature for all polymers. The resulting change of kinetics is more pronounced in the PB-PEG copolymers than in the linear PB. According to the relative sequence of crystallization kinetics, two temperature regions can be distinguished, where a threshold temperature is located at 70 °C. For the low temperature region (*T*_iso_ ≤70 °C), incorporating PEG grafts to main chain accelerates crystallization with respect to linear PB. For example, at 50 °C, reaching half crystallization requires around 20 s for PB, which is almost twice that of the PB-PEG copolymers. Then, in the high temperature region (*T*_iso_ > 70 °C), temperature elevation depresses PB-PEG crystallization more than PB.

It is known that crystallization is complemented by nucleation and growth steps. Figures S5 and S6 compare the nucleation densities after crystallization at 60 °C and 85 °C, respectively, which are representative of low and high temperature regions, respectively. As shown in Figure S5, the incorporation of a TMS branch is already able to increase the nucleation density significantly, while the presence of long PEG grafts enhances nucleation further. Considering the results in Figure 1, that PB-TMS crystallization capability is lower than PB, it could be inferred that the linear growth rate is smaller in PB-TMS than PB. This also implies that the growth rate is further reduced in PB-PEG copolymers, as observed in the long-chain branched PLA samples [41]. This implies that in PB-TMS and PB-PEG copolymers, nucleation density was increased but the linear growth rate was decreased with respect to PB. It should be noted that the POM results of Figures S5 and S6 cannot support the comparison among the different PEG graft lengths studied in this work. Then, whether increasing PEG graft length from 750 to 4000 g/mol enhances nucleation density or not requires further investigation.

### 3.3. Melting Behavior

**Figure 5:**
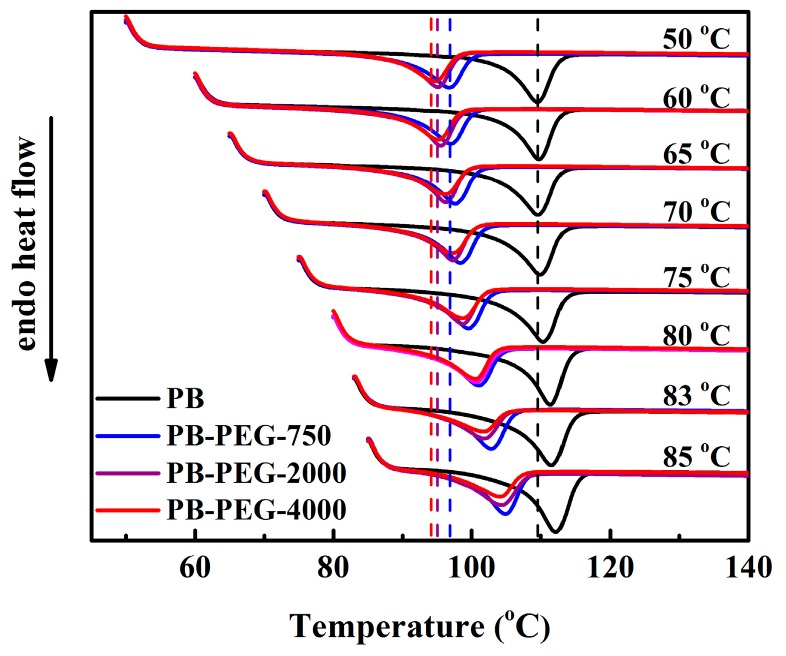
DSC melting curves of PB and PB-PEG crystallites prepared by isothermal crystallization at different temperatures. The thermal protocol was illustrated in Figure S3b.

Figure 5 gives the DSC melting curves of the crystallites after isothermal crystallization at different temperatures, in order to examine the thermal stability of the crystallites. Although the crystallizations happened at identical temperatures, the melting temperatures of PB-PEG copolymers are all lower than that of PB crystals. Especially, PB-PEG form II crystallized at 85 °C melts at *T*_m_ = 104.9 °C, which is even lower than *T*_m_ = 109.5 °C of PB crystallized at 50 °C.

**Figure 6:**
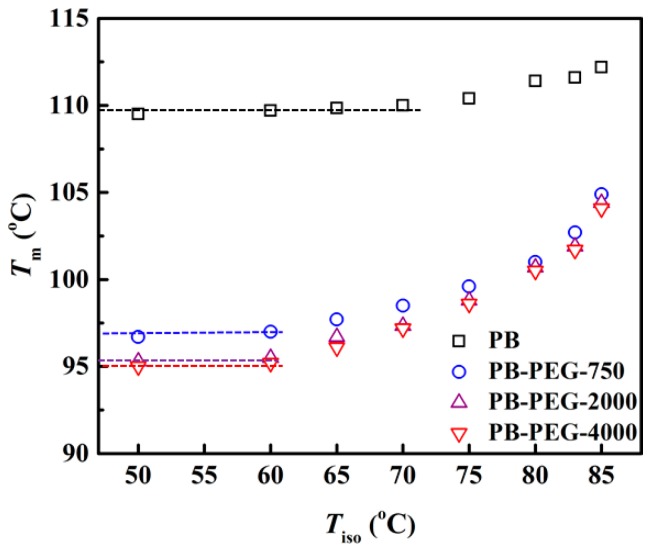
The melting temperatures of the PB and PEG-grafted polybutene-1 crystallites prepared by isothermal crystallization at different temperatures.

Figure 6 gives the melting temperatures *T*_m_ of PB and PB-PEG as a function of crystallization temperature *T*_iso_. For all polymers, there is one critical crystallization temperature, below which the melting temperature is constant for different crystallization temperatures. This is associated to the recrystallization process that occurs during heating [50]. For PB, the critical crystallization temperature is 70 °C, which agrees well with the value reported by Wang et al. [51]. However, the introduction of PEG grafts decreases the critical temperature to 60 °C.

### 3.4. Polymorphic Form II to Form I Phase Transition

**Figure 7:**
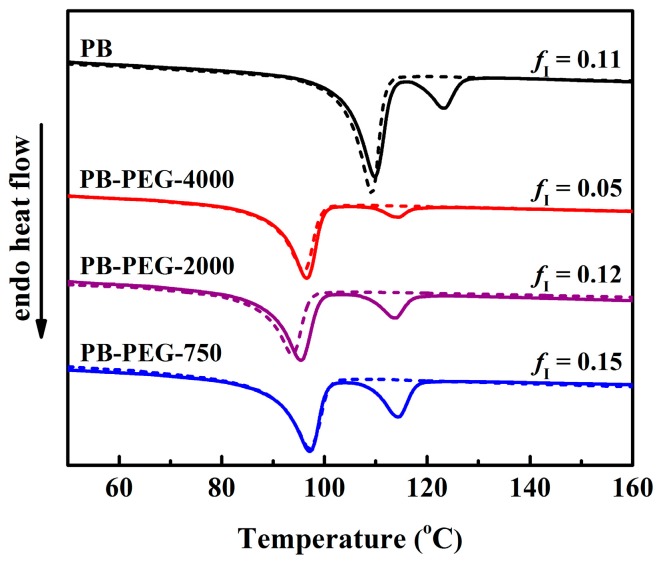
DSC melting curves of the PB and grafted PB-PEG copolymers after annealing at 25 °C for 50 h (solid line), where initial form II was crystallized isothermally at 60 °C. The dashed line indicates the melting curves of form II without annealing.

For polybutene-1 homopolymer and copolymers, the melt-crystallized form II tends to transform into the stable form I. The crystallites generated by the above isothermal crystallization were annealed at 25 °C for 50 h to allow for II-I phase transition. Without phase transition, the preparation of pure form II crystallites was also confirmed by the XRD measurements (Figure S7). Figure 7 shows the DSC melting curves of partially transformed samples for the crystallites prepared by isothermal crystallization at 60 °C. As shown by the dashed lines, the initial form (form II) has a single endothermic peak. As soon as the II-I phase transition happens, extra melting peaks emerge at the high temperatures, which corresponds to the transformed form I [18,34]. It is clear that the II-I phase transition happens for all of the copolymers studied in this work. When the transition duration is fixed, the fraction of transformed form I corresponds to the transition rate. Fractions of transformed form I in total crystallites (*f*_I_) are quantified using the following equation:

(3) $$f_{I}=\frac{\Delta H_{m\_I}/\Delta H_{I}^{0}}{\Delta H_{m\_I}/\Delta H_{I}^{0}+\Delta H_{m\_II}/\Delta H_{II}^{0}}$$

where $\Delta H_{m\_I}$ and $\Delta H_{m\_II}$ are the measured melting enthalpies of forms I and II, respectively (Figure S8). $\Delta H_{I}^{0}$ and $\Delta H_{II}^{0}$ represent the ideal melting enthalpies of forms I and II, which are 141 and 62 J/g, respectively [49]. For 25 °C and 50 h, the determined fractions of transformed form I are also given in Figure 7. In PB, the fraction of form I transformed at 25 °C for 50 h is 0.11. It can be seen that the II-I transition rate of grafted PB-PEG copolymers strongly depends on the PEG length. PB-PEG-750 has the largest transition rate of 0.15 after annealing for 50 h. When increasing the PEG length to *M*_n_ = 2000 and 4000 g/mol, the transition rate decreases to 0.12 and 0.05, respectively. The extension of the PEG graft increases the steric hindrance and retards phase transition.

**Figure 8:**
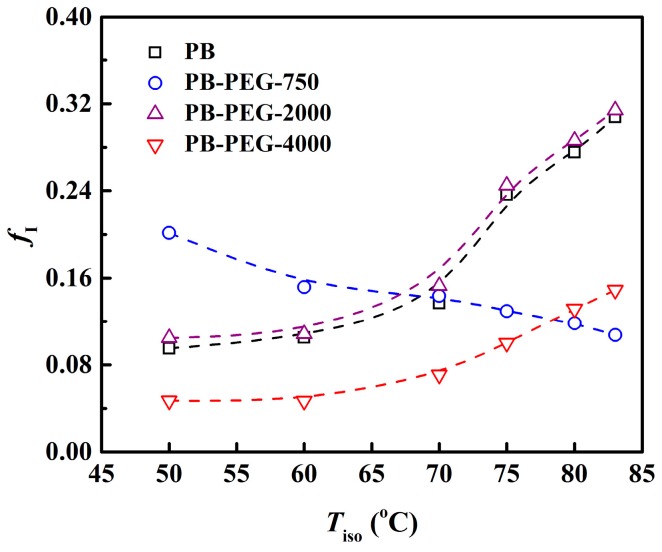
Fraction of transformed form I (*f*_I_) in the PB and PEG-grafted copolymers as a function of isothermal crystallization temperature. The thermal protocol is illustrated in Figure S3c.

Figure 8 summarizes the transition rates of PB and PEG-grafted copolymers for a broader crystallization temperature (DSC melting data given in Figure S9). Note that a very fast cooling rate of 50 °C/min was utilized to prevent cooling crystallization and allow isothermal crystallization at a broad temperature range from 50 to 83 °C. It can be seen that the temperature dependence of the phase transition rate varies vastly with different lengths of PEG grafts. For linear PB, the transition rate often decreases with a decreasing crystallization temperature. This is due to the reason that when the transition temperature is fixed at 25 °C, the decrease of crystallization temperature reduces the internal thermal stress caused, which is the driving force for phase transition. Similarly, in PB-PEG copolymers with long grafts of *M*_n_ = 2000 and 4000 g/mol, transition rates decrease with reducing crystallization temperatures. As the PEG grafts were increased from *M*_n_ = 2000 to 4000, the transition rate was reduced. This is probably because of the steric effect of the PEG grafts, which retards the necessary helical and positional adjustment of the crystalline stems within the crystals [15,31]. However, in PB-PEG-750, with the shortest PEG grafts, the transition rate increases with decreasing crystallization temperature, similar to the PB homopolymer with *M*_w_ = 77 kg/mol [33]. In summary, the II-I phase transition can be tuned by the control of the PEG length.

## 4. Conclusions

In this work, comonomer Ph-TMS was copolymerized with butene-1 to obtain the trimethylsilyl and carbon-carbon triple bond groups, which can be employed to further prepare grafted butene-1 copolymers with different PEG grafts (*M*_n_ = 750, 2000 and 4000 g/mol). The crystallization, melting, and phase transition were systematically investigated. The DSC results showed that for cooling crystallization, the incorporation of a TMS branch significantly decreases the peak temperature of cooling crystallization, by more than 20 °C with respect to the linear polybutene-1 homopolymer. However, PEG-grafted copolymers have a greater crystallization temperature than PB-TMS, which is even higher than that of PB. This indicates that presence of PEG grafts enhances copolymer crystallization, although PEG grafts also disturb the regularity of main chains. Then, the results of isothermal crystallization demonstrate that there is a critical temperature of 70 °C, below which the crystallization of PB-PEG copolymers is always faster than that of PB. In the temperature region above 70 °C, PB crystallization may exceed that of PB-PEG copolymers, depending on whether the increase of nucleation density can compensate the reduction of growth rate. Moreover, the DSC heating results display that the melting temperatures of the PB-PEG copolymer are lower than that of the PB.

When form II, crystallized at 60 °C, is annealed at 25 °C, PB-PEG-750, with *M*_n_ = 750 g/mol grafts, exhibits an accelerated transition rate that is faster than the homopolymer PB. However, a negative correlation between the transition rate and crystallization temperature was identified in PB-PEG-750 for a broad temperature range of 50 °C to 83 °C. The transition rate of PB-PEG-2000 is very close to that of PB. As the PEG length was further increased to 4000 g/mol, the transition rate was retarded, which is probably due to steric effect.

## Figures and Tables

**Table 1:** Molecular characteristics of the homopolymer, copolymer, and PEG-grafted copolymers.

Sample Code	Incorp. ^a^ (mol %)	*M*_n_ of PEG (Da)	*M*_w_^b^ (10^5^ Da)	*M*_w_/*M*_n_^b^
PB	0	None	1.98	2.38
PB-TMS	0.49	None	2.08	2.84
PB-PEG-750		750	2.59	2.50
PB-PEG-2000		2000	2.77	2.44
PB-PEG-4000		4000	3.05	2.93

^a^ Comonomer incorporation (mol%) was determined by ^1^H-NMR spectra at 120 °C with C_2_D_2_Cl_4_ as a solvent. ^b^ Determined by gel permeation chromatography (GPC) at 150 °C in 1,2,4-C_6_Cl_3_H_3_ versus polystyrene standards.

**Table 2:** Crystallization temperature (*T_c_*) and crystallinity (*X*_c_) of the PB, PB-TMS, and PB-PEG copolymers.

Sample Code	PB	PB-TMS	PB-PEG-4000	PB-PEG-2000	PB-PEG-750
*T*_c_ (°C)	58.3	36.5	63.2	63.4	68.1
*X*_c_ (%)	41.0	29.3	39.1	38.5	39.1

## Supplementary Materials

The following are available online at https://www.mdpi.com/2073-4360/11/5/837/s1. NMR results, GPC results, kinetics of isothermal crystallization at other temperatures, POM results, fitting method of DSC data, DSC melting curves of samples crystallized at different temperatures after annealing at 25 °C for 50 h.
