# Supplementary material for: Thermal Analysis of Crystallization and Phase Transition in Novel Polyethylene Glycol Grafted Butene-1 Copolymers
Source: Polymers (Basel). 2019 May 8;11(5):837. doi: 10.3390/polym11050837 (PMC6572506; doi:10.3390/polym11050837)
Supplement: Supplementary file 1 [file polymers-11-00837-s001.pdf]

Supplementary Materials

Thermal Analysis of Crystallization and Phase Transition in Novel Polyethylene Glycol Grafted Butene-1 Copolymers

Chuanbin An,[1] Yulian Li,[1] Yahui Lou,[1] Dongpo Song,[1] Bin Wang,[1] Li Pan,[1] Zhe Ma,[1,*] and Yuesheng Li[1,2]

1 Tianjin Key Laboratory of Composite and Functional Materials, and School of Materials Science and Engineering, Tianjin University, Tianjin 300072, P. R. China; ancb0306@tju.edu.cn (C. A.); liyulian@tju.edu.cn (Y. L.); yhlou@tju.edu.cn (Y. L.); dongpo.song@tju.edu.cn (D. S.); binwang@tju.edu.cn (B. W.); lilypan@tju.edu.cn (L. P.); zhe.ma@tju.edu.cn (Z. M.); ysli@tju.edu.cn (Y. L.)

2 Collaborative Innovation Center of Chemical Science and Engineering (Tianjin), Tianjin 300072, P. R. China; ysli@tju.edu.cn (Y. L.)

* Correspondence: zhe.ma@tju.edu.cn; Tel.: +86-22-2740-2887

Received: date; Accepted: date; Published: date

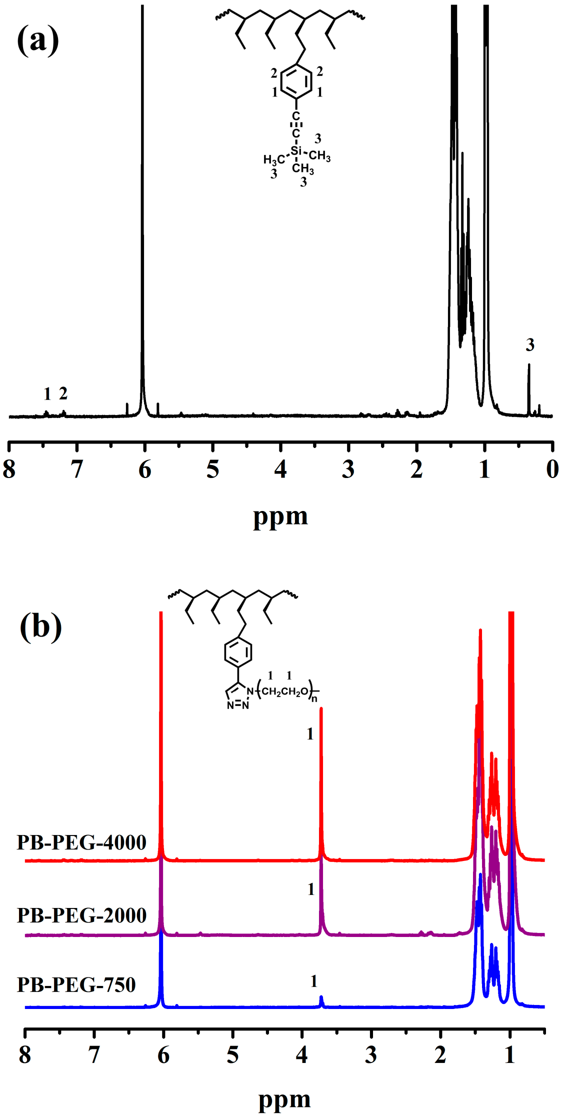

Figure S1. ^{1}H-NMR spectra of copolymer PB-TMS and PEG-grafted copolymers with different PEG molecular weights.

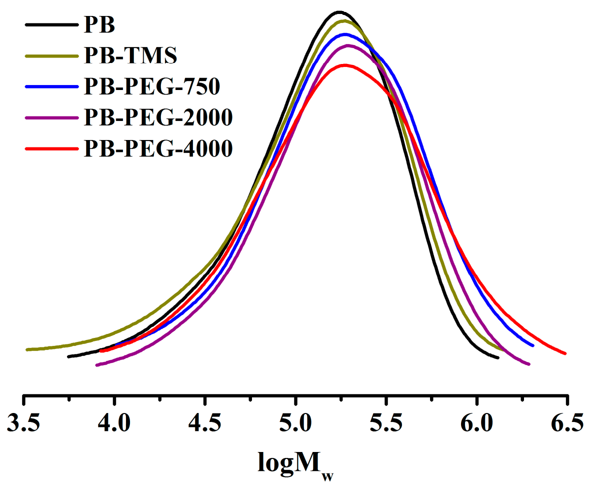

Figure S2. GPC profiles of homopolymer, copolymer PB-TMS and PEG-grafted copolymers.

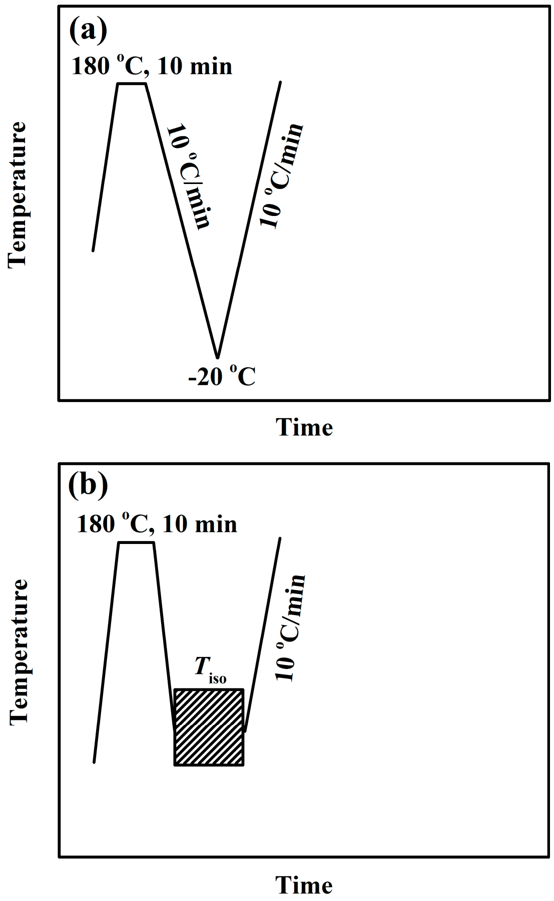

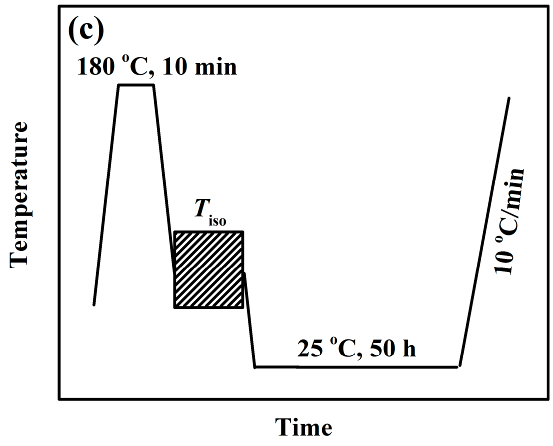

Figure S3. Schematic illustrations of thermal protocols applied for (a) cooling crystallization, (b) isothermal crystallization at different temperatures, and (c) phase transition at 25°C.

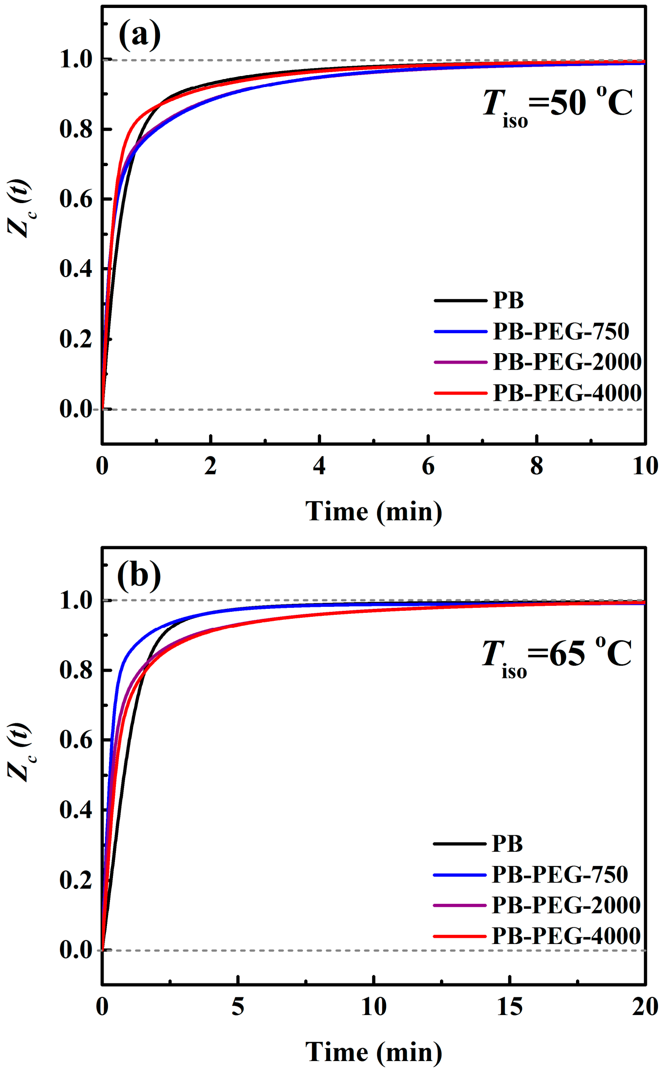

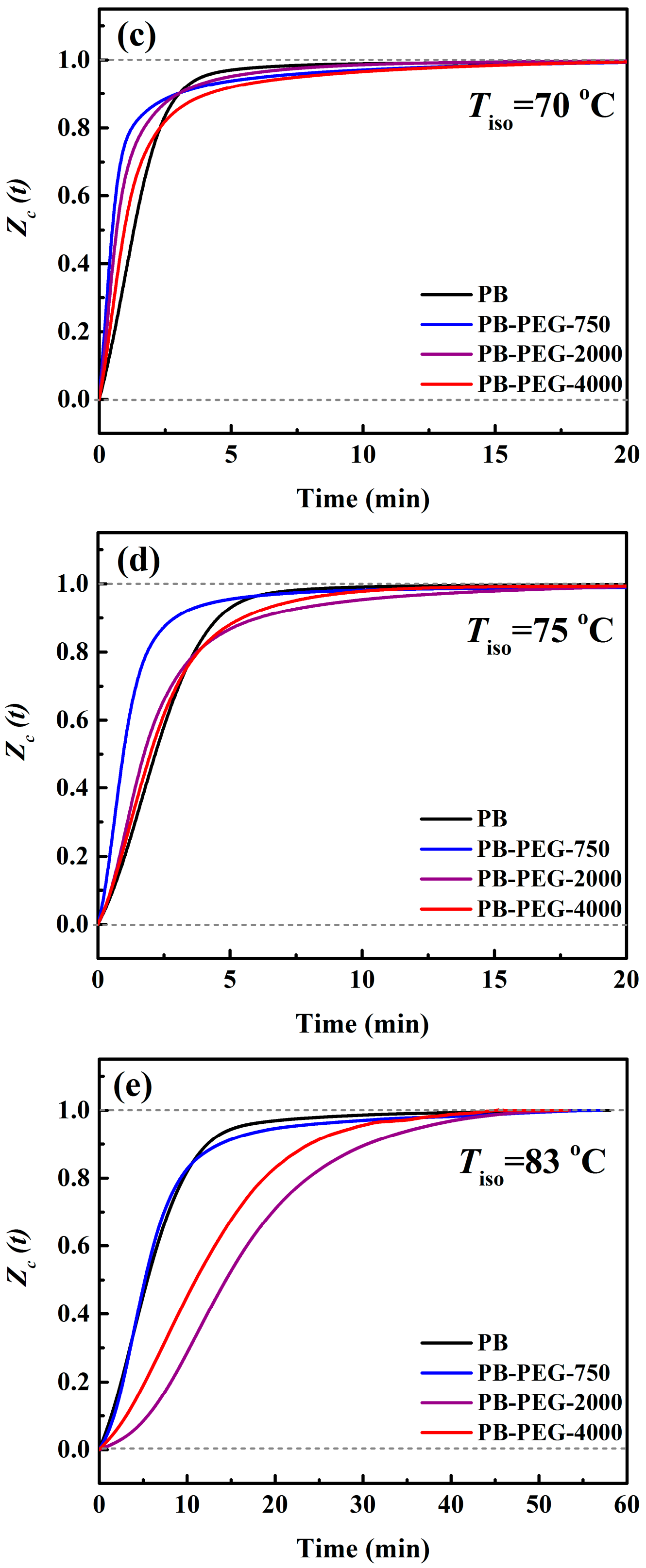

Figure S4. Kinetics of isothermal crystallization at (a) 50, (b) 65, (c) 70, (d) 75, and (e) 83°C.

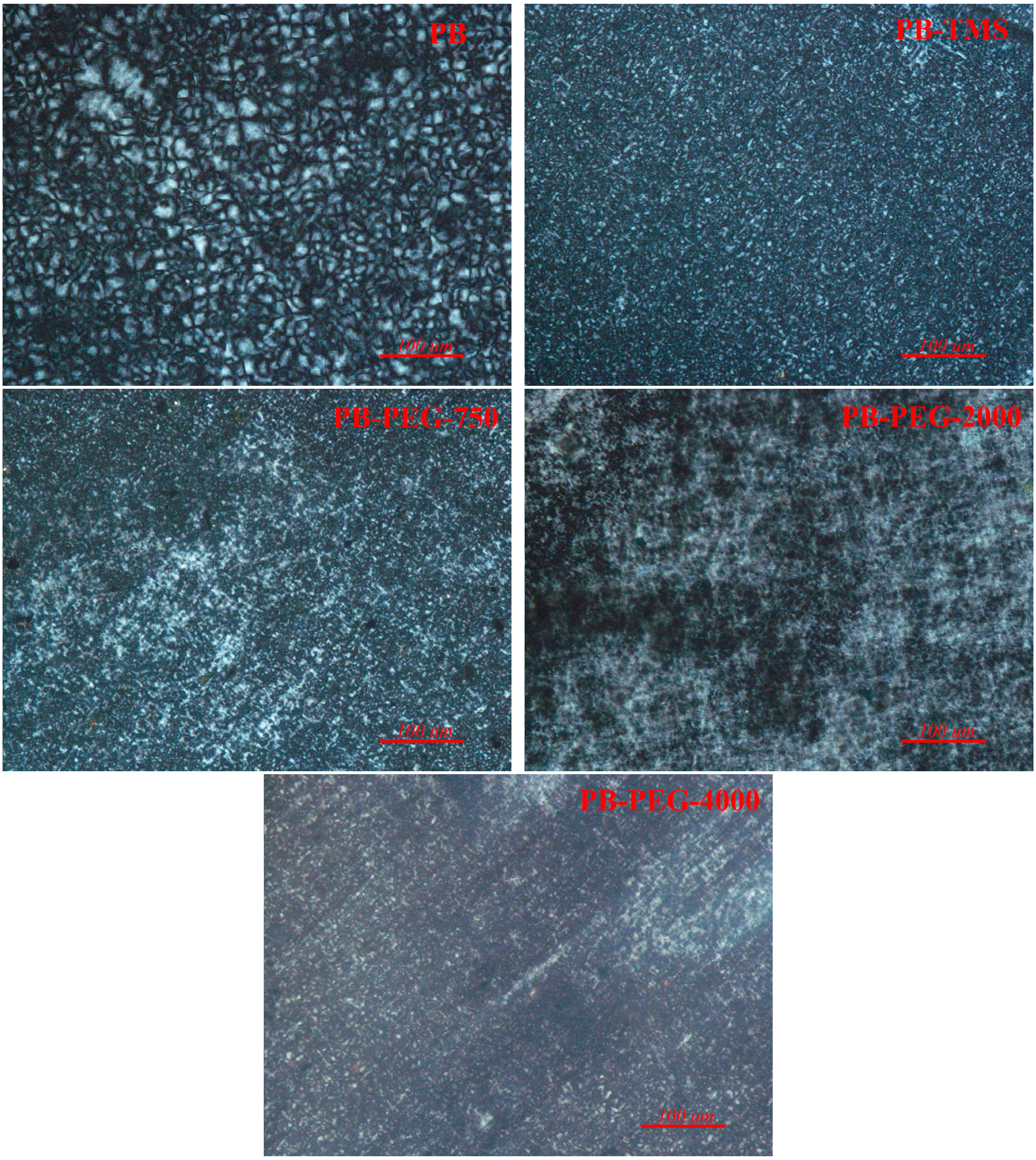

Figure S5. Polarized optical micrographs of homopolymer, copolymer PB-TMS and PEG-grafted copolymers crystallized at 60°C.

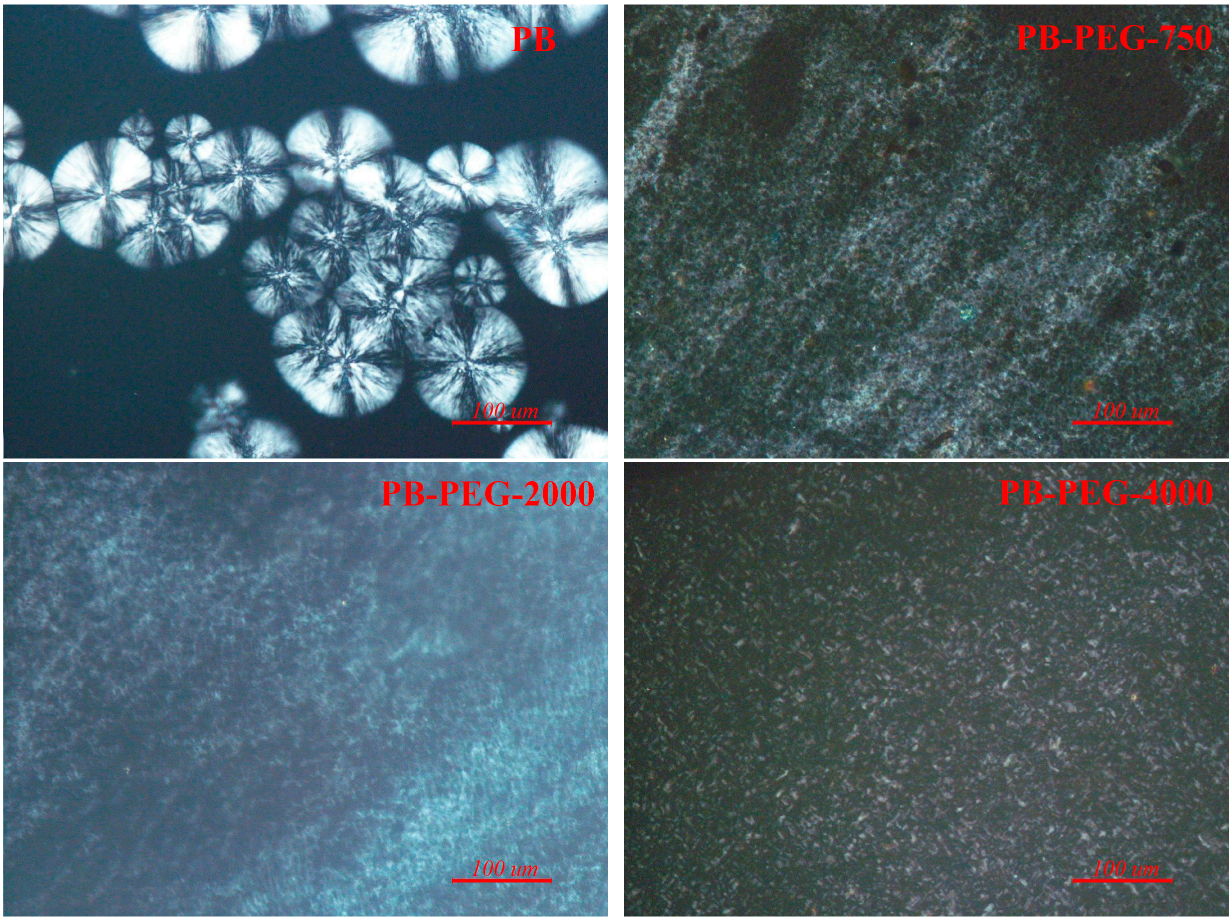

Figure S6. Polarized optical micrographs of homopolymer and PEG-grafted copolymers crystallized at 85°C for their $t_{1/2}$.

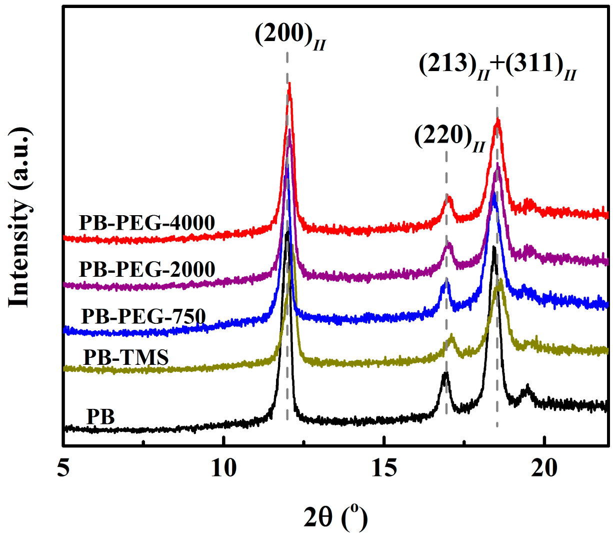

Figure S7. XRD curves of PB, PB-TMS, and PB-PEG copolymers crystallized during cooling at 10°C/min.

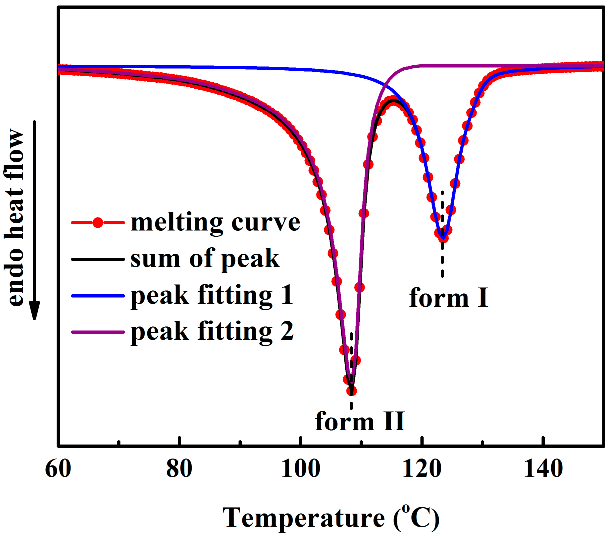

Figure S8. Fitting method to determine the melting entropies of residual form II and transformed form I.

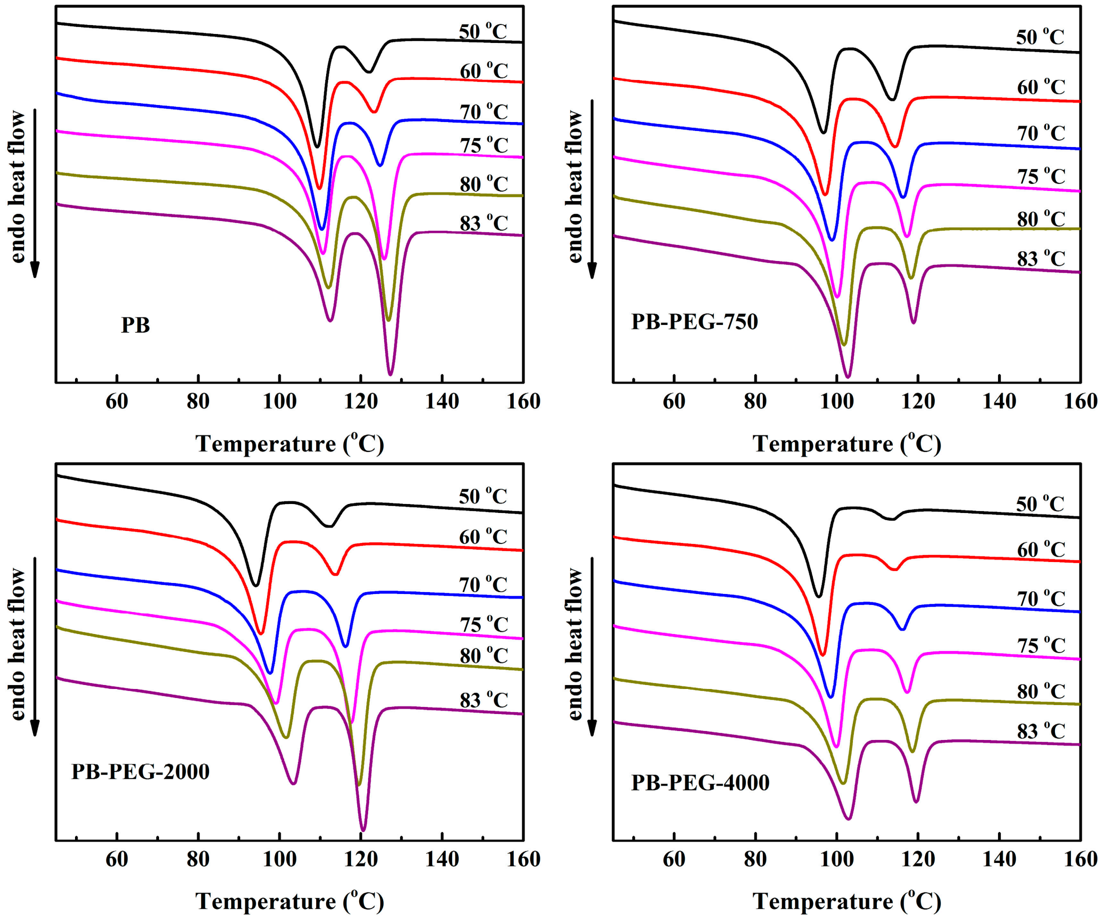

Figure S9. DSC heating curves of homopolymer and PEG-grafted copolymers after annealing at 25°C for 50 h as a function of the isothermal crystallization temperature.
